# Cerebral gas embolism in a patient without right-to-left shunt after robotic partial nephrectomy

**DOI:** 10.1186/s40981-025-00793-w

**Published:** 2025-05-24

**Authors:** Mori Satori, Mitsuhiro Matsuo, Yoshinori Ikehata, Hiroshi Kitamura, Tomonori Takazawa

**Affiliations:** 1 Department of Anesthesiology, University of Toyama, Toyama, Japan; 2 Department of Urology, University of Toyama, Toyama, Japan

**Keywords:** Carbon dioxide, Case report, Gas embolism, Paradoxical embolism, Nephrectomy

## Abstract

**Background:**

Cerebral gas embolism is a rare but serious complication of laparoscopic surgeries, the risk of which is increased by the presence of right-to-left shunt. A case of cerebral gas embolism after robotic partial nephrectomy is presented.

**Case presentation:**

A 71-year-old man underwent robotic partial nephrectomy. During tumor resection, end-tidal CO₂ (ETCO₂) decreased from 42 to 34 mmHg, followed by a decrease in mean arterial pressure (MAP) to < 65 mmHg and oxygen saturation (SpO₂) to 95%. Postoperatively, he exhibited delayed emergence from anesthesia and left conjugate gaze deviation. Neuroimaging revealed cerebral gas embolism. A bubble test performed by a cardiologist under positive pressure ventilation ruled out right-to-left shunt. Despite postoperative treatment, the patient became bedridden with severe neurological sequelae.

**Conclusions:**

Cerebral gas embolism can occur during robotic procedures even without right-to-left shunt. Anesthesiologists must promptly recognize intraoperative signs of this complication and initiate timely interventions to prevent severe complications.

**Supplementary Information:**

The online version contains supplementary material available at 10.1186/s40981-025-00793-w.

## Background

Robotic partial nephrectomy is widely used for the resection of renal cancers [1]. Similar to laparoscopic surgery, the use of carbon dioxide (CO_2_) insufflation in robotic-assisted procedures can lead to complications, including subcutaneous emphysema, hypercapnia, and gas embolism [2]. Subclinical gas embolization, detected by transesophageal echocardiography (TEE) during laparoscopic surgery, has a high incidence rate, ranging from 38 to 100% [3]. However, clinically relevant gas embolism is rare, with an estimated incidence of approximately 0.15% in laparoscopic procedures [4].

Paradoxical embolization occurs when embolic material passes from the right to the left side of the heart through an intracardiac or intrapulmonary shunt, such as a patent foramen ovale, potentially leading to cerebral infarction [5]. In fact, cases of cerebral infarction following laparoscopic surgery have been reported in patients with right-to-left shunts [6, 7]. Here, we report a case of cerebral gas embolism following robotic partial nephrectomy in a patient without evidence of a right-to-left shunt.

## Case presentation

**Fig. 1:**
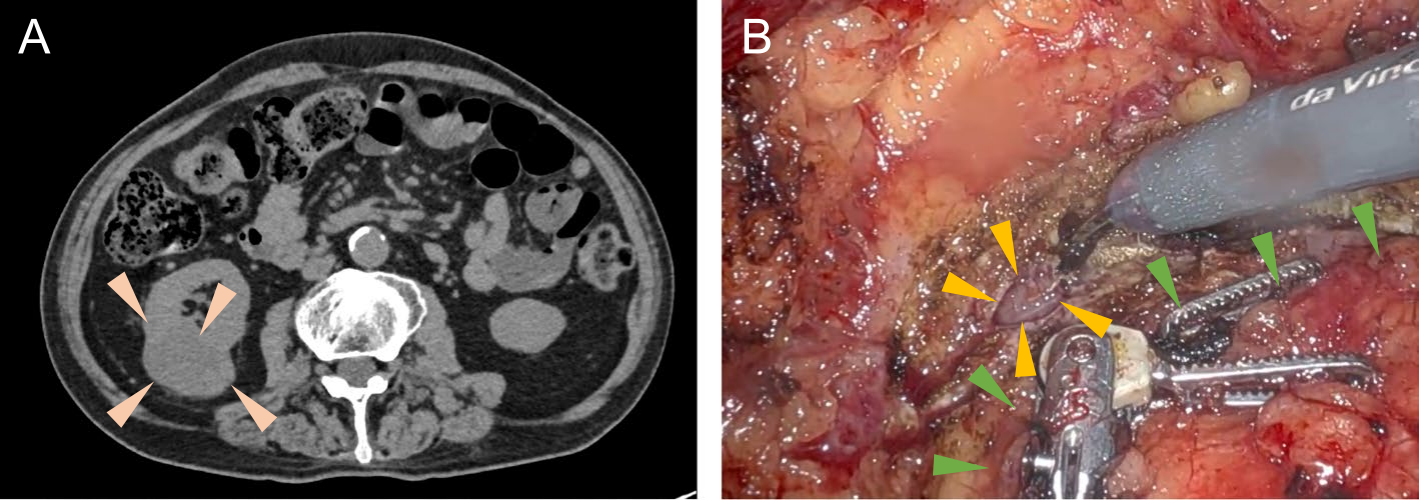
Preoperative and intraoperative images of the renal tumor. **A** Preoperative abdominal CT scan showed a tumor located at the lower pole of the right kidney. **B** Intraoperative photograph showing the injured vein. The robotic surgery was performed using the da Vinci Xi surgical system®. The tumor (green arrowheads) was retracted with a fenestrated grasper, and its margins were dissected using monopolar curved scissors. During the procedure, although no injury to large veins, such as the inferior vena cava and renal vein, occurred, a dilated vein located at the margin of the renal tumor (yellow arrowheads) was injured during tumor resection, and remained open for 22 min

A 71-year-old Japanese man (162 cm tall, weighing 54.5 kg) with independence in activities of daily living, presented with a 41.5 × 30.5 × 31.5 mm solid tumor in the right kidney that was clinically diagnosed as renal cell carcinoma (Fig. 1A), for which he was scheduled for robotic partial nephrectomy. Preoperative evaluation, including electrocardiogram, chest X-ray, and blood tests, showed no abnormalities, with normal liver and renal function. His medical history included well-controlled type 2 diabetes, hypertension, and dyslipidemia.

Upon his arrival to the operating room (8:22 a.m.), his mean arterial pressure (MAP) was 106 mmHg, heart rate (HR) was 62 beats/min, and peripheral oxygen saturation (SpO_2_) was 97% on room air. General anesthesia was induced with remifentanil 0.3 μg/kg/min, propofol 70 mg, and rocuronium 30 mg. Noradrenaline was used at 0.02 to 0.03 μg/kg/min throughout surgery. A catheter was placed in the radial artery for invasive arterial pressure monitoring. Following endotracheal intubation, mechanical ventilation was initiated with a tidal volume of 450 mL, respiratory rate of 10 breaths/min, positive end-expiratory pressure of 5 cmH_2_O, and an inspiratory oxygen concentration of 40%. After creation of the pneumoperitoneum, the respiratory rate was increased to 14 breaths/min. Neuromuscular blockade was monitored using an acceleromyogram at the abductor pollicis muscle to maintain a train-of-four ratio of zero and a post-tetanic count of zero or higher. Surgery was performed in the left lateral decubitus position. The pneumoperitoneum pressure was maintained at 10 mmHg using the Airseal iFS insufflator (CONMED, Utica, NY, USA) throughout the surgery.

**Fig. 2:**
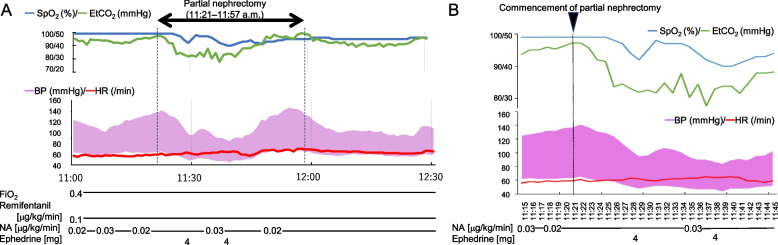
Intraoperative changes in vital signs during partial nephrectomy. **A** Partial nephrectomy was performed from 11:21 a.m. to 11:57 a.m. without clamping of the renal vein. Immediately after the partial nephrectomy was commenced, the patient’s ETCO_2_, blood pressure and SpO_2_ decreased. It took approximately 1 h for the vital signs to recover to pre-resection levels. **B** Enlarged graph showing the vital sign changes between 11:15 and 11:45 as depicted in **A**. FiO_2_, fraction of inspired oxygen; NA, noradrenaline

Surgery was performed using a retroperitoneal approach. At 11:21 a.m., the right renal artery was clamped, and partial nephrectomy was commenced (Supplementary video S1). The attending urologists opted not to clamp the renal vein at its site of drainage into the inferior vena cava. At this point, the patient’s MAP was 87 mmHg, HR was 60 beats/min, SpO₂ was 99%, and end-tidal carbon dioxide (ETCO₂) was 41 mmHg. However, at 11:26 a.m., his ETCO₂ abruptly decreased from 42 to 34 mmHg (Fig. 2), and the surgeon reported difficulties in the procedure due to respiratory variability. In response, the anesthesiologist reduced the tidal volume from 450 mL to 300 mL. The patient’s SpO₂ decreased to 95% by 11:28 a.m., followed by a decrease in MAP to below 65 mmHg, prompting intravenous ephedrine administration. MAP improved above 65 mmHg by 11:45 a.m., and ETCO₂ recovered above 40 mmHg by 11:46 a.m. Partial nephrectomy was completed at 11:57 a.m. However, the patient’s SpO₂ did not recover to above 96% until 12:26 p.m. A review of the surgical video performed after the completion of the procedure revealed that at 11:22 a.m., a dilated vein at the tumor margins was injured and remained open until 11:44 a.m. This open vein was eventually closed as part of routine hemostasis (Fig. 1B).

**Fig. 3:**
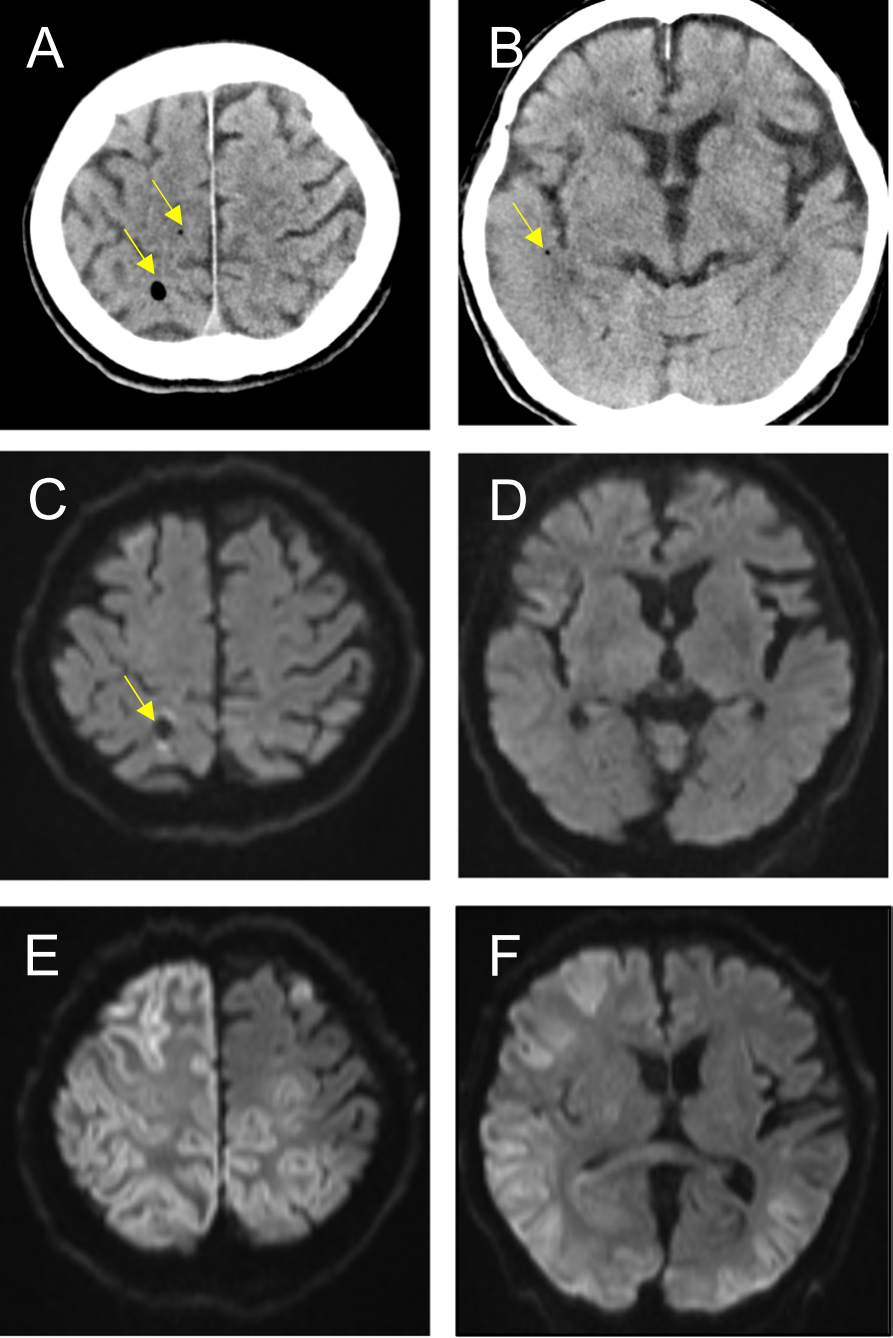
Evidence of cerebral gas embolism on neuroimaging. **A**, **B** A non-enhanced CT scan taken approximately 2 h after the start of partial nephrectomy. Air bubbles (arrows) were seen along the vessels of the cerebral surface. **C**, **D** MRI, including diffusion-weighted imaging sequences, performed approximately 3 h after the start of partial nephrectomy showed slight high intensity areas along the brain surface. **E**, **F** MRI performed on POD 7 showed extensive cerebral infarcts over the cerebral hemispheres bilaterally, consistent with the left conjugate eye deviation, epilepsy, and delayed emergence from anesthesia

At the end of surgery (12:52 p.m.), the patient’s vital signs were stable, but delayed emergence from anesthesia was noted. At this time, left conjugate eye deviation raised the concern of epileptic seizures, prompting a head computed tomography (CT) scan at 2:15 p.m. CT revealed intracranial gas distributed along the cortical arteries of the right hemisphere, leading to a diagnosis of cerebral gas embolism (Fig. 3). A neurosurgical consultation was obtained, and magnetic resonance imaging (MRI) performed at 3:14 p.m. showed high-signal intensity areas on diffusion-weighted imaging (DWI) in some cortical regions, with normal T2 images.

At 5:00 p.m., hyperbaric oxygen therapy was commenced with a protocol of compression to 2.5 atmospheres absolute over 15 min, maintaining pressure for 60 min, followed by decompression over 15 min. On postoperative day (POD) 2, disappearance of the intracranial air bubbles was confirmed on head CT scan. However, hyperbaric oxygen therapy was discontinued due to development of treatment-resistant epileptic seizures.

On POD 7, head MRI revealed extensive cerebral infarction throughout the right cerebral hemisphere, the left posterior cerebral artery region, and the posterior circulation. A tracheostomy was performed by otolaryngologists on POD 13 to manage airway needs. Intravenous edaravone 30 mg was administered every 12 h until POD 14. On POD 15, a cardiologist conducted a bubble test using TEE under positive pressure ventilation, but no bubbles were detected in the left ventricle, ruling out intracardiac or intrapulmonary shunts. Additionally, a chest CT reviewed by pulmonologists confirmed the absence of pulmonary arteriovenous malformations. Although rehabilitation improved his neurological status to E4 V2M4 on the Glasgow Coma Scale, he remained non-communicative and with severe left hemiparesis. On POD 73, he was transferred to a rehabilitation hospital for further care.

## Discussion

Here, we presented a case of a patient without a right-to-left shunt, who developed severe cerebral gas embolism following robotic partial nephrectomy. A bubble test performed by a cardiologist under positive pressure ventilation ruled out the presence of such a shunt. A similar case has been previously reported in a patient without an intracardiac shunt who developed cerebral infarction after laparoscopic hepatic surgery, where a pulmonary shunt related to cirrhosis was suspected to have contributed to the massive cerebral gas embolism [4].

Even in patients without an intracardiac shunt, intraarterial carbon dioxide is detected in about 2% of cases during laparoscopic partial hepatectomy [3]. However, the entry of carbon dioxide bubbles into cerebral arteries does not always result in cerebral stroke [8, 9], suggesting that the volume of bubbles is critical to the development of serious complications. Animal studies have shown that the detection rate of bubbles within the left ventricle is dependent on the amount of intravenous gas [10, 11]. Thus, even in patients without a right-to-left shunt, the risk of cerebral gas embolism increases as the amount of gas entering the vein increases.

Early detection of venous gas embolism is crucial for preventing cerebral gas embolism. TEE is a sensitive method of detecting venous gas, but the presence of venous gas does not necessarily indicate symptomatic gas emboli [3]. We did not perform a TTE immediately after we suspected a gas embolus because TTE after the onset of symptoms does not lead to a cure. Severe venous gas embolism is often accompanied by tachycardia in animal studies [12], and with a decrease in ETCO_2_ in clinical reports [13, 14]. Mild to moderate venous gas emboli, on the other hand, are also known to cause a transient increase in ETCO₂, as the gas may dissolve into the bloodstream and be exhaled before leading to hemodynamic compromise [15]. In the present case, a decline in ETCO_2_ was observed a few minutes after the commencement of tumor resection, followed by hypotension and desaturation. These findings suggest that a massive air embolization occurred in our patient. Therefore, anesthesiologists should consider the possibility of cerebral gas embolism when encountering vital sign changes suggestive of venous gas embolism during procedures involving potential vascular injury. In case of the development of venous injury, reducing pneumoperitoneal pressure can help minimize gas entry into the venous system [3]. Additionally, ventilation strategies aimed at increasing central venous pressure may also help to reduce the pressure gradient between the pneumoperitoneum and central venous pressure [3]. If cerebral gas embolism is diagnosed, hyperbaric oxygen therapy should be administered immediately [16].

In robot-assisted laparoscopic partial nephrectomy, urologists often clamp only the renal arteries while resecting the tumor. Animal studies have shown that additional clamping of the renal vein leads to lower intraoperative renal tissue oxygen saturation [17] and a reduction in postoperative glomerular count compared to clamping the artery alone [18]. Although avoiding renal vein clamping is expected to help preserve postoperative renal function, clinical studies have yet to demonstrate a clear advantage in terms of long-term renal outcomes in real-world settings [19, 20]. Given that renal vein clamping may reduce the risk of gas embolism and bleeding, it may be reasonable for anesthesiologists to consider suggesting renal vein clamping during tumor resection to urologists.

In this case, DWI performed approximately 3 h after the start of the nephrectomy revealed a slight subcortical cerebral infarct, but MRI on POD 7 showed a more extensive infarct (Fig. 3). Subcortical infarction is characteristic of cerebral infarction secondary to gas embolism [21, 22]. The pathogenesis is thought to involve leukocyte activation, disruption of the blood-brain barrier, and obstruction of cerebral blood flow by gas [21]. Although cerebral infarction is typically detectable on DWI within 3 h of onset [23], gas embolism-related infarcts might not appear on imaging for several hours [22]. As demonstrated in this case, MRI findings might initially be inconsistent with clinical symptoms following gas embolism.

## Conclusion

We presented a case of cerebral gas embolism during robotic partial nephrectomy in a patient without evidence of a right-to-left shunt. Anesthesiologists should remain attentive, as cerebral gas embolism can occur during any laparoscopic procedure, even in the absence of a right-to-left shunt. Careful interpretation of neuroimaging is essential, given the potential discrepancies between imaging findings and clinical presentation.

## Supplementary Information

Supplementary Material 1: Supplementary video S1. To-and-fro blood flow probably through the injured vein. At 00:51, the vein at the tumor margin appears to be incised using monopolar curved scissors. Notably, blood outflow can be observed at timestamps 01:11, 01:20, 01:24, and 01:28. This flow pattern occurred approximately 14 times per minute, likely corresponding with the mechanical ventilation rate.

## Data Availability

Not applicable.

## Abbreviations

CO_2_: Carbon dioxide
CT: Computed tomography
DWI: Diffusion-weighted imaging
ETCO₂: End-tidal CO₂
HR: Heart rate
MAP: Mean arterial pressure
MRI: Magnetic resonance imaging
SpO₂: Oxygen saturation
TEE: Transesophageal echocardiography
